# A novel attention-based hybrid CNN-RNN architecture for sEMG-based gesture recognition

**DOI:** 10.1371/journal.pone.0206049

**Published:** 2018-10-30

**Authors:** Yu Hu, Yongkang Wong, Wentao Wei, Yu Du, Mohan Kankanhalli, Weidong Geng

**Affiliations:** 1 State Key Lab of CAD&CG, College of Computer Science and Technology, Zhejiang University, Hangzhou, China; 2 Smart Systems Institute, National University of Singapore, Singapore, Singapore; 3 School of Computing, National University of Singapore, Singapore, Singapore; Chinese Academy of Sciences, CHINA

## Abstract

The surface electromyography (sEMG)-based gesture recognition with deep learning approach plays an increasingly important role in human-computer interaction. Existing deep learning architectures are mainly based on Convolutional Neural Network (CNN) architecture which captures spatial information of electromyogram signal. Motivated by the sequential nature of electromyogram signal, we propose an attention-based hybrid CNN and RNN (CNN-RNN) architecture to better capture temporal properties of electromyogram signal for gesture recognition problem. Moreover, we present a new sEMG image representation method based on a traditional feature vector which enables deep learning architectures to extract implicit correlations between different channels for sparse multi-channel electromyogram signal. Extensive experiments on five sEMG benchmark databases show that the proposed method outperforms all reported state-of-the-art methods on both sparse multi-channel and high-density sEMG databases. To compare with the existing works, we set the window length to 200ms for NinaProDB1 and NinaProDB2, and 150ms for BioPatRec sub-database, CapgMyo sub-database, and csl-hdemg databases. The recognition accuracies of the aforementioned benchmark databases are 87.0%, 82.2%, 94.1%, 99.7% and 94.5%, which are 9.2%, 3.5%, 1.2%, 0.2% and 5.2% higher than the state-of-the-art performance, respectively.

## Introduction

The surface electromyogram signal [1] records muscle’s information by putting non-invasive surface sEMG electrodes on the skin. The electrical activity recorded by sEMG electrodes allows us to develop human-computer interface (HCI) system which has been employed in four major areas [2]: **(1)** Assistive technology (e.g., myoelectric controlled prosthesis [3], wheelchair [4] and assistive robots [5]), **(2)** Rehabilitative technology (e.g., sEMG-driven Exoskeletons [6] and serious games [7, 8]), **(3)** Input technology (e.g., armbands and MCI [9]), and **(4)** Silent speech recognition [10]. Among these applications, sEMG-based hand gesture recognition plays an important and fundamental role for computers or assistive devices to understand human body language.

The traditional sEMG-based gesture recognition framework consists of data preprocessing, feature extraction, feature selection and gesture classification. Among these stages, feature extraction and gesture classification are two important stages in sEMG-based gesture recognition framework. Therefore, researchers have focused on presenting discriminative feature sets with domain knowledge [11–15], as well as employing conventional machine learning algorithms to classify hand gestures [16–19]. These works often require excessive parameter tuning and rich domain knowledge.

In recent years, deep learning techniques achieve promising performance in various fields [20–23] and provide a new perspective to analyze sEMG for hand gestures recognition. Inspired by the excellent performance of deep learning techniques, the Convolutional Neural Network (CNN) has been exploited for sEMG-based gesture recognition [24–29]. Park and Lee [24] proposed a CNN model with adaptive feature learning to improve the inter-subject accuracy. The CNN-based sEMG gesture recognition was studied in [25] which achieved comparable performance with traditional methods on the NinaPro database. Geng *et al*. [26, 27] presented a new CNN architecture for instantaneous sEMG images and the recognition accuracies are 77.8% for 52 gestures, 99.5% for 8 gestures and 89.3% for 27 gestures on three sEMG benchmark databases. Du *et al*. [28] designed a semi-supervised deep CNN framework which employed data glove to provide auxiliary information.

Overall speaking, existing deep learning methods for sEMG-based gesture recognition are mainly based on CNN architecture. However, the sEMG is a form of sequential data by its nature. In the field of video classification and human activity analysis, the hybrid CNN-RNN architecture has obtained good performance when compared with pure CNN-based approaches [30–33]. Aiming at modeling the temporal information better than conventional CNN-based architectures, we investigate a hybrid CNN-RNN architecture for sEMG-based gesture recognition to capture both spatial and temporal information. Moreover, attention mechanism has been applied to the proposed hybrid CNN-RNN architecture for it has proven successful in sequential data modeling (e.g., machine translation [34], image caption generation [35] and speech recognition [36]).

The main contributions of this work are twofold:

1. We propose an attention-based hybrid CNN-RNN architecture for sEMG-based gesture recognition, which models both the spatial and temporal information of sEMG and focuses on the subsegments which contain more discriminative information for gesture recognition. Compared with the CNN module of proposed architecture which only models the spatial information, it improves the recognition accuracies from 83.5% to 84.8% on the first sub-database of NinaPro (denoted as NinaProDB1), from 73.4% to 74.8% on the second sub-database of NinaPro (denoted as NinaProDB2), from 83.9% to 92.5% on a sub-database of BioPatRec (denoted as BioPatRec26MOV), from 97.7% to 99.7% on a sub-database of CapgMyo (denoted as CapgMyo-DBa) and from 92.1% to 94.9% on the csl-hdemg, respectively.
2. Motivated by the idea of signal image [37], we present a new sEMG image representation method based on a feature vector for sparse multi-channel electromyogram signals. It rearranges feature vectors based on the classical feature set Phinyomark [13], and is evaluated on three sparse multi-channel sEMG databases using the CNN, hybrid CNN-RNN and attention-based hybrid CNN-RNN frameworks, respectively. The results show that the proposed sEMG image representation method is superior to the existing sEMG image representation method. The improvements are at least 2.0% for NinaProDB1, 7.4% for NinaProDB2 and 1.6% for BioPatRec26MOV.

The remainder of the paper is organized as follows. Firstly, we review related works on sEMG-based gesture recognition methods, hybrid CNN and RNN architectures and the attention mechanism. Secondly, we introduce the proposed attention-based hybrid CNN-RNN architecture for sEMG-based gesture recognition, and describe the details of the new feature vector based sEMG image representation methods. Thirdly, we show the experimental results on five benchmark sEMG databases. Finally, we draw the conclusion and discuss our future work.

## Related work

In this section, we present related works on sEMG-based gesture recognition methods, the hybrid CNN-RNN architectures and attention mechanism in the literature.

The handcrafted features and traditional machine learning classifiers have been extensively researched in early sEMG-based gesture recognition frameworks. Existing sEMG-based handcrafted features can be divided into three categories [38]: time domain, frequency domain, and time-frequency domain features. Many researchers focused on presenting new sEMG features based on their domain knowledge [14, 15] or analyzing existing features to propose new feature sets [13, 39]. Traditional machine learning classifiers have been employed to recognize sEMG-based gestures, such as k-Nearest Neighbor (kNN) [16], Linear Discriminate Analysis (LDA) [17, 40, 41], Hidden Markov Model (HMM) [18, 42], and Support Vector Machine (SVM) [14, 19]. The SVM is the most popular classifier in early sEMG-based gesture recognition frameworks. Patricia *et al*. [19] utilized Geodesic Flow Kernel with SVM classifier to classify 10 gestures. Doswald *et al*. [14] applied SVM classifier with Pearson VII Universal Kernel to recognize 5 gestures. As electromyogram signal is sequential data, HMM is suitable for modeling electromyogram signal with hidden information. Yun *et al*. [42] used HMM classifier to create a sign language recognition system based on sEMG.

The CNN architecture is the most widely used deep learning technique for sEMG-based gesture recognition, which can be divided into two categories based on different evaluation methods. The first study focuses on improving recognition accuracy of intra-session evaluation [25, 26]. Atzori *et al*. [25] constructed sEMG images which contain both spatial and temporal information and trained a CNN model to extract high-level features. Geng *et al*. [26] provided a novel CNN model to extract spatial information from the instantaneous sEMG images and achieved state-of-the-art performance. The second study is devoted to the difference between sessions or subjects [24, 27]. Park and Lee [24] draw adaptation method into CNN model to learn better features for inter-subject evaluation. Du *et al*. [27] applied domain adaptation based on the GengNet [26] to improve the inter-session accuracy. Zhai *et al*. [29] extracted useful information from the sEMG spectrogram to form sEMG images and a CNN-based architecture was employed to model the relationship between sEMG images and gesture labels.

The RNN architecture has been applied for sEMG-based hand problems, such as pose estimation [43, 44] and sEMG feature extraction [45, 46]. Hioki and Kawasaki [43] presented a neural network with recurrent structure to estimate finger joint angles using sEMG signal. Quivira *et al*. [44] proposed a sEMG-based hand pose estimation method using RNN with LSTM cells. It constructed a model for predicting the hand joint kinematics through sEMG signals and captured hand pose kinematics accurately. Amor *et al*. [45] applied Myo armband to collect sEMG signals for sign language recognition and employed the RNN architecture to extract features from sequential data for analyzing sign language gestures. Shin *et al*. [46] exploited an RNN architecture with three LSTM layers to extract features from sEMG and Inertial Measurement Unit (IMU) signals for Korean sign recognition.

The hybrid CNN-RNN architecture has obtained good performance in recognition of video and wearable sensors. Ebrahimi Kahou *et al*. [30] presented a hybrid CNN-RNN architecture for facial expression analysis. The CNN and RNN module of the architecture were trained separately. Wu *et al*. [31] designed a hybrid deep learning framework to extract spatial, short-term and long-term features which consist of two hybrid CNN-RNN architecture and a regularized fusion layer. Ordóñez and Roggen [32] proposed a deep hybrid CNN-RNN for activity recognition with multimodal wearable sensors. This architecture provided a natural sensor fusion and modeled temporal information of the activity. Wang *et al*. [33] recommended a novel CNN-LSTM model to solve both gesture recognition and pose estimation problem with only RGB videos. The CNN block was employed to extract spatial features from each frame, and the proposed sequentially supervised LSTM (SS-LSTM) used auxiliary knowledge instead of the class label to supervise learning process.

The attention mechanism has been injected into RNN architectures for performance enhancement in many application scenarios. Dzmitry *et al*. [34] proposed an RNN encoder-decoder model with attention for machine translation. It jointly learned for alignment and translation and achieved significant performance improvement over the basic encoder-decoder method. Kelvin *et al*. [35] introduced two attention-based image caption generators and the results on three benchmark databases showed the effectiveness of attention. Chorowski *et al*. [36] presented a novel attention-based neural speech recognition architecture. The performance was comparable to that of the traditional methods on the TIMIT dataset. Song *et al*. [47] provided an end-to-end LSTM network with spatial and temporal attention modules for skeleton-based human action recognition. The recognition accuracies on two benchmark databases outperformed other state-of-the-art methods.

As mentioned above, the hybrid CNN-RNN architecture has been successfully applied to activity recognition based on video and wearable sensors. The attention mechanism is also an effective way to enhance the performance of RNN architecture.

Since the electromyogram signal is noisier than other wearable sensor signals [32], we extract the Phinyomark feature set [13] of each channel to generate new sEMG images and employ deep neural network to extract useful information between each channel. However, the generated sEMG image is a monochrome image and has much smaller pixel resolutions than normal images or video frames. We carefully fine-tune parameters of each layer and add locally-connected layer [26] to our proposed attention-based hybrid CNN-RNN architecture which has been applied in sEMG-based gesture recognition for the first time.

## Methods

### Attention-based hybrid CNN-RNN architecture

**Fig 1:**
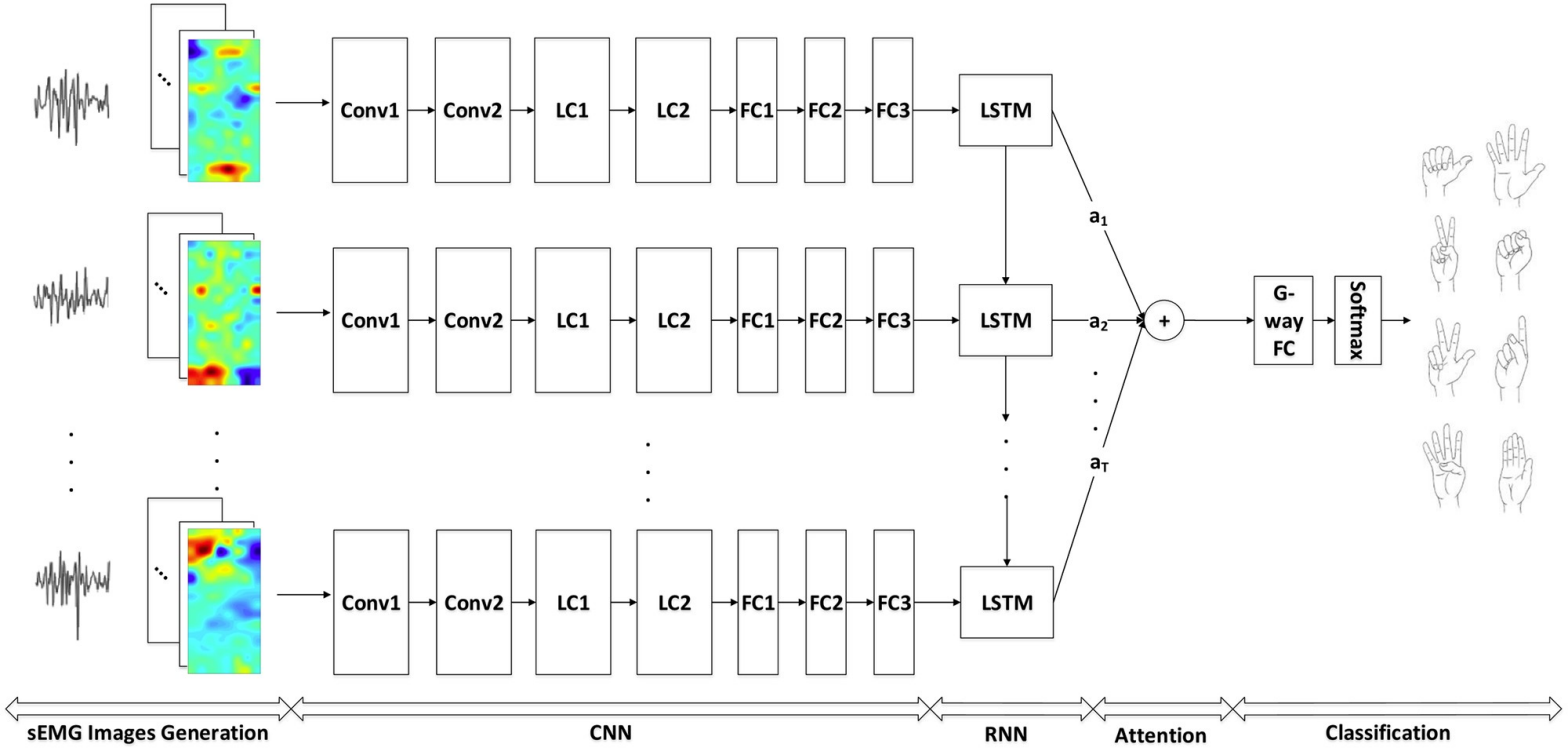
Proposed attention-based hybrid CNN-RNN architecture for sEMG-based gesture recognition.

The attention mechanism has been proposed in deep learning to learn from the way a human perceives the real-world that paying attention to different regions [48]. The motor unit action potential (MUAP) generates and propagates along the muscle fibers [26, 49] and muscles have varying importance in contributing to different hand movements [50]. If the learned classification model can effectively capture these important factors of the involved muscles, it may bring performance improvements on sEMG-based gesture recognition. The attention framework in deep learning usually models the importance inside the training data through weights and has been successfully applied to various tasks, such as image caption generation [35], speech recognition [36], sentiment analysis [51] and *etc*. Therefore, we focus on how to embed the attention mechanism into the classification model and accordingly propose a novel attention-based hybrid CNN-RNN architecture for sEMG-based gesture recognition (see Fig 1).

The proposed architecture models both the spatial-temporal information and importance of the input electromyogram signals and the layers configuration of the proposed network are shown in Table 1.

**Table 1:** The layers configuration of proposed attention-based hybrid CNN-RNN.

Layers	Name	Configurations	Modules
1	**Conv1**	64 kernels, kernel size (3 × 3)	CNN
2	**Conv2**	64 kernels, kernel size (3 × 3)	
3	**LC1**	64 kernels	
4	**LC2**	64 kernels	
5	**FC1**	512 outputs	
6	**FC2**	512 outputs	
7	**FC3**	128 outputs	
8	**LSTM1**	LSTM, 512 hidden unit outputs	RNN
9	**Attention**		Attention
10	**G-way FC**		Classification
11	**Softmax**		

Each sample recorded from *C* electrodes with *L* frames is denoted as ***X***. We first use sliding window method to split ***X*** into subsegments which are denoted as {***X*_1_, *X*_2_, …, *X*_*T*_**}, where *T* (*T* ≤ *L*) is the number of subsegments and also time steps of RNN. Each subsegment ***X*_*t*_**, ∀*t* = 1, 2, …, *T* has *N* frames with the size of 1 × *C*. Then, the ***X*_*t*_** is converted into an image ***I*_*t*_** with size (*N* × *W* × *H*), where *W* × *H* = *C*, *W* and *H* are width and height of the image, respectively. The detailed image representation method can be seen in section. Given the converted images {***I*_1_, *I*_2_, …, *I*_*T*_**}, CNN is applied as feature extractor to transform them into feature vectors {***F*_1_, *F*_2_, …, *F*_*T*_**}. The CNN model consists of seven layers. The first two layers are convolutional layers with 64 3 × 3 kernels, followed by two locally-connected layers. The locally-connected layer with 64 1 × 1 kernels is employed to extract local features of the sEMG image. Batch normalization [52] is used for each of the layers mentioned before reducing the internal covariate shift. The last three layers are all fully-connected layers with batch normalization, and a dropout with the probability of 0.5 is applied to the first two fully-connected layers. For the sequence modeling stage, each RNN unit has a dropout with the probability of 0.5 and 512 hidden units, followed by a *G*-way fully-connected layer and a softmax classifier. *G* is the number of gestures to be recognized. The final label is decided by average-pooling of the softmax outputs.

The recurrent neural network contains feedback loops and encodes contextual information of a temporal sequence. Given the input sequence {***F*_1_, *F*_2_, …, *F*_*T*_**} (feature vectors extracted from CNN model), the hidden states ***h*_*t*_** and outputs ***y*_*t*_** can be calculated as follows:

$$\begin{array}{ccc} h_{t} & = & H(W_{ih}F_{t}+W_{hh}h_{t-1}+b_{h})\\ y_{t} & = & W_{ho}h_{t}+b_{o} \end{array}$$ (1)

where ***W*_*ih*_, *W*_*hh*_, *W*_*ho*_** are weight matrices between input, hidden and output layers. As standard RNN suffers from gradient vanishing or exploding problem, long short-term memory (LSTM) [53] has been proposed to alleviate this issue. Each LSTM unit consists of input gate, output gate, forget gate and cell, and the calculating relations between them are as follows:

$$\begin{array}{c} \begin{array}{ccc} i_{t} & = & \delta (W_{i}\left[h_{t-1},F_{t}\right]+b_{i})\\ f_{t} & = & \delta (W_{f}\left[h_{t-1},F_{t}\right]+b_{f})\\ o_{t} & = & \delta (W_{o}\left[h_{t-1},F_{t}\right]+b_{o})\\ {\hat{c}}_{t} & = & tanh(W_{c}\left[h_{t-1},F_{t}\right]+b_{c})\\ c_{t} & = & f_{t}⊙c_{t-1}+i_{t}⊙{\hat{c}}_{t}\\ h_{t} & = & o_{t}⊙tanh\left(c_{t}\right) \end{array} \end{array}$$ (2)

where *δ* is the logistic sigmoid function and ***i***, ***f***, ***o*** and ***c*** are input, forget, ouput gate and cell activation.

An attention layer [51] is employed to enhance the performance of hybrid CNN-RNN architecture. Its calculation formula is as follows.

$$\begin{array}{c} \begin{array}{ccc} M_{t} & = & tanh(W_{h}h_{t})\\ \alpha_{t} & = & softmax(w^{T}M_{t})\\ r & = & \sum_{t=1}^{T}\alpha_{t}h_{t} \end{array} \end{array}$$ (3)

where *h*_*t*_ is the output of the t-th hidden unit of RNN module, *α*_*t*_ is the t-th attention weight, ***W*_*h*_** and ***w*^*T*^** are weighted matrices and *r* is the output of attention module. The output ***r*** is followed by a G-way fully-connected layer and a softmax classifier.

#### Loss function

The loss function of attention-based hybrid CNN-RNN architecture is:

$$\begin{array}{c} loss=\alpha \cdot loss_{\text{attention}}+\beta \cdot loss_{\text{target}}+\lambda \cdot {\left||w|\right|}^{2} \end{array}$$ (4)

where the first term is the attention loss, the second term is the target replication loss [54] and the last term is the regularization term. The *α*, *β* and λ are three weight parameters.

$$\begin{array}{c} loss_{\text{attention}}=\frac{1}{T}\cdot l\left(g_{1}\left(X\right),y\right) \end{array}$$ (5)

$$\begin{array}{c} l\left(g_{1}\left(X\right),y\right)=-\sum_{i=1}^{G}1_{i}\left(y\right)log\;g_{1}{\left(X\right)}^{i} \end{array}$$ (6)

$$\begin{array}{c} g_{1}\left(X\right)=f_{s}\left(f_{a}\left(f_{h}\left(X_{1}\right),f_{h}\left(X_{2}\right),\ldots ,f_{h}\left(X_{T}\right)\right)\right) \end{array}$$ (7)

where ***X*** is the electromyogram signal to be recognized, *y* is the ground-truth label, and *T* is the number of time steps of RNN. *G* is the number of gestures to recognize, *g*_1_(***X***)^*i*^ is the *i*-th dimension of *g*_1_(***X***) and **1**_*i*_() is the indicator function. *f*_*h*_, *f*_*a*_ and *f*_*s*_ stand for the hybrid CNN-RNN architecture, attention module and the last softmax layer, respectively.

$$\begin{array}{c} loss_{\text{target}}=\frac{1}{T}\sum_{t=1}^{T}l(g_{2}\left(X_{t}\right),y) \end{array}$$ (8)

$$\begin{array}{c} l\left(g_{2}\left(X_{t}\right),y\right)=-\sum_{i=1}^{G}1_{i}\left(y\right)log\;g_{2}{\left(X_{t}\right)}^{i} \end{array}$$ (9)

$$\begin{array}{c} g_{2}\left(X_{t}\right)=f_{s}\left(f_{h}\left(X_{t}\right)\right) \end{array}$$ (10)

where ***X***_*t*_ is the *t*-th subsegment of ***X*** and *g*_2_(***X***_*t*_)^*i*^ is the *i*-th dimension of *g*_2_(***X***_*t*_). *f*_*h*_ and *f*_*s*_ stand for the hybrid CNN-RNN architecture and the last softmax layer.

### Image representation from temporal electromyogram signals

Existing sEMG databases can be divided into two categories: sparse multi-channel sEMG database [55] and high-density sEMG database [26, 56]. We generate sEMG images for both sparse multi-channel and high-density sEMG databases. As mentioned in [26], we convert a segment of electromyogram signal into a sEMG image which has the same dimensions (i.e., color channel, width, and height) as the RGB image.

An intuitive sEMG image representation method is to use the placement of electrodes and each electrode can be regarded as a pixel of sEMG images. It is a feasible sEMG image representation method for high-density sEMG databases csl-hdemg and CapgMyo-DBa, as electromyogram signals are collected by a grid of sEMG electrodes. The detailed image representation procedure is described as follows. The input is a segment of electromyogram signal of the high-density electromyogram signal with size *L* × *W* × *H*, where *L* is the number of frames, *W* is rows of the array electrode and *H* is columns of the array electrode. The raw signal is converted into a sEMG image with size *L* × *W* × *H*, where *L* is the number of color channels of the sEMG image, *W* is the width of sEMG image and *H* is the height of sEMG image. The sEMG image size of csl-hdemg and CapgMyo-DBa are *L* × 24 × 7 and *L* × 16 × 8, respectively.

However, for sparse multi-channel sEMG database, the number of electrodes is limited and the placement is sparse. Inspired by the image representation method used in [37] for human activity recognition with accelerometer and gyroscope, there are six image representation methods for raw electromyogram signal, namely **raw-image1**, **raw-image2**, **signal-image1**, **signal-image2**, **activity-image1** and **activity-image2**. The input of the sEMG image representation methods is a segment of electromyogram signal of NinaProDB1 with size *L* × *C*, where *L* is the number of frames, *C* is the number of signal channels and *C* = 10 for NinaProDB1. The detailed sEMG image representation methods are described as follows.

1. The **raw-image1** is obtained by transforming the input into a sEMG image with size *L* × 1 × 10, where *L* is the number of color channels of the sEMG image, 1 is the width of sEMG image, and 10 is the height of sEMG image.
2. The **raw-image2** [25] is obtained by transforming the input into a sEMG image with size 1 × *L* × 10, where 1 is the number of color channels of the sEMG image, *L* is the width of sEMG image, 10 is the height of sEMG image, and width × height = signal channels.
3. The **signal-image1** [37] is formed by rearranging the data of each signal channel in [37] with size *L* × 1 × 51.
4. The **signal-image2** [37] is formed by the same procedure as **signal-image1** with size 1 × *L* × 51.
5. The **activity-image1** [37] is generated by FFT transformation of signal-image1 with size *L* × 1 × 51.
6. The **activity-image2** [37] is generated by FFT transformation of signal-image2 with size 1 × *L* × 51.

**Fig 2:**
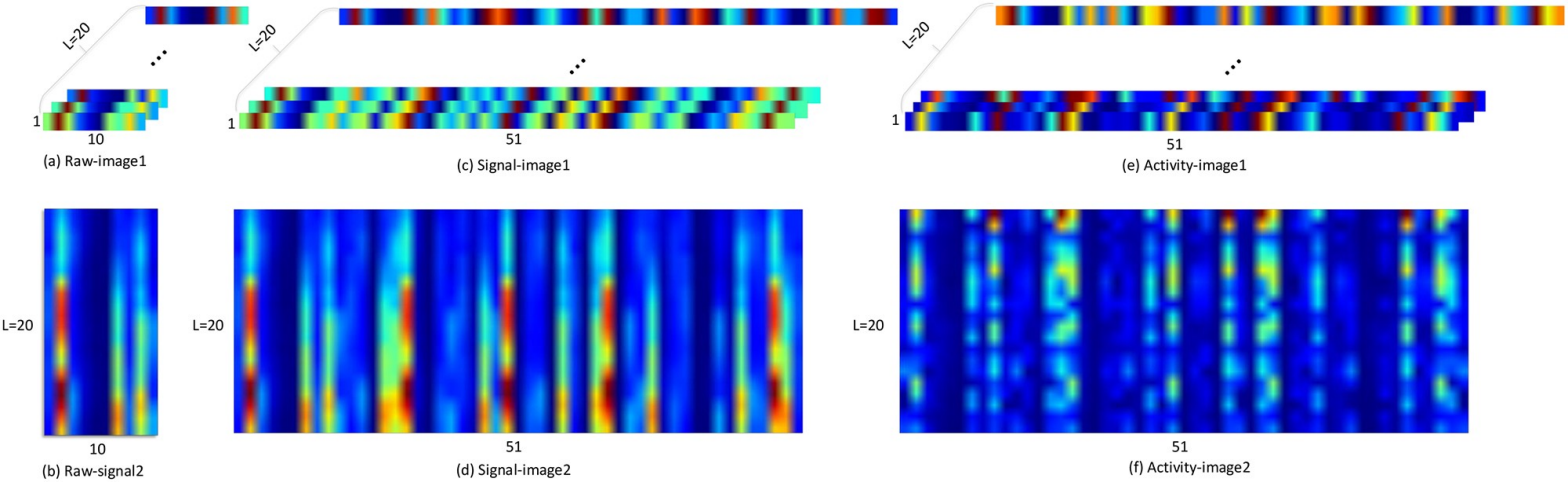
Six raw signal based sEMG image representation methods.

We evaluate the six sEMG image representation methods using the existing CNN architecture [26] on NinaProDB1. Firstly, the electromyogram signal is segmented by the sliding window with 200ms length and converted into six sEMG images which can be found in Fig 2. Then, the training set and test set are the same as those described in the experimental setup. Finally, GengNet [26] is employed to respectively extract useful information of the six sEMG images. The classification accuracy in Table 2 shows that the signal-image method is a feasible sEMG image representation method to improve recognition accuracy for sparse multi-channel electromyogram signal. The signal-image method achieves higher classification accuracy than the raw-image method for signal-image method contains more information between different channels. The activity-image methods perform the worst in three image representation methods because of the FFT transform which may cause time-domain information loss.

**Table 2:** Comparison of gesture recognition accuracy with various image representation methods on NinaProDB1. Here, we employ the GengNet [26], and the sliding window length is fixed at 200ms for all experiments.

sEMG Image	Classification Accuracy
raw-image1	83.5%
raw-image2	82.9%
signal-image1	84.9%
signal-image2	79.8%
activity-image1	78.1%
activity-image2	72.8%
feature-signal-image1	**86.3%**

The feature extraction plays a significant role in traditional sEMG-based gesture recognition methods and many classical feature sets have achieved good performance [11, 13, 15]. Therefore, we want to generate a new sEMG image based on the traditional feature vector. The most obvious idea is to flatten all the feature vectors of different channels into one vector with size feature dimension = signal channels × feature vector dimension and conduct a sEMG image with size 1 × 1 × feature dimension which is denoted as feature-flatten-image. We have evaluated this sEMG image representation method on the NinaProDB1, and the recognition accuracy is 81.9% which is lower than that of raw signal based sEMG images (i.e., “raw-image1” and “signal-image1”). Inspired by the good performance of signal-images, we propose a new sEMG image representation method “feature-signal-image1” which makes full use of the traditional feature vector and achieves better performance among existing sEMG image representation methods.

## Experiments and results

In this section, we first delineate the experimental setup, followed by the performance comparisons between proposed architecture and state-of-the-art methods on five sEMG benchmark databases. Secondly, we discuss the effectiveness of attention mechanism. Then, the results of different image representation methods are presented. Finally, we evaluate and discuss the impacts of various parameters of the architecture on recognition accuracy.

### Experimental setup

In this work, we follow the experimental process which consists of data acquisition, preprocessing, segmentation and gesture recognition. The proposed architecture is implemented using MxNet [57], and the evaluations are carried out on five sEMG benchmark databases, namely NinaProDB1, NinaProDB2, BioPatRec26MOV, CapgMyo-DBa and csl-hdemg. The details summary of all database are shown in Table 3.

**Table 3:** Details of five sEMG benchmark databases.

Database	Subjects	Gestures	Sessions	Trials	Number of electrodes	Sampling rate (Hz)
NinaproDB1	27	52	1	10	10	100
NinaProDB2	40	50	1	6	12	2000
BioPatRec26MOV	17	26	1	3	8	2000
CapgMyo-DBa	18	8	1	10	128	1000
csl-hdemg	5	27	5	10	192	2048

The first and second sub-database of NinaPro database [55] are denoted as NinaProDB1 and NinaProDB2, respectively. NinaProDB1 contains a total of 52 gestures from 27 subjects, including 9 wrist movements, 8 hand postures, 12 finger movements and 23 grasping and functional movements. The electromyogram signal is filtered by a low-pass Butterworth filter [26, 55]. NinaProDB2 collects 50 gestures from 40 subjects, including 23 grasping and functional movements, 9 wrist movements, 8 hand postures, 9 finger force patterns and the rest position. The electromyogram signal is filtered by a low-pass Butterworth filter [26, 55] and is downsampled to 100HZ which is NinaProDB1’s sampling rate.

A subset of BioPatRec toolbox is available online [58] (denoted as BioPatRec26MOV), which collects 26 hand movements from 17 subjects using 8 sEMG electrodes. The duration of the contraction is based on a contraction time percentage, which is set to the default value 0.7 [15].

The first sub-database of CapgMyo database [26] (denoted as CapgMyo-DBa), which contains 8 hand gestures from 18 subjects and each gesture performed 10 trials. The electromyogram signal is band-pass filtered [26] in the data collection.

The csl-hdemg database [56] contains 27 finger gestures from 5 subjects, where each subject was recorded 5 sessions and performed each gesture 10 trials in each session. The electromyogram signal is rectified [59] and filtered by a low-pass Butterworth filter in pre-processing.

Given the preprocessed electromyogram signal, we decompose it into small segments using the sliding window strategy with overlapped windowing scheme to fully utilize the computing capacity of the system [60]. The window length must be shorter than 300ms [11] to satisfy real-time usage constraints. To compare our proposed method with previous works, we follow the segmentation strategy in previous studies. The window length is fixed to 150ms and 200ms for NinaProDB1, 200ms for NinaProDB2, 50ms and 150ms for BioPatRec26MOV, 40ms and 150ms for CapgMyo-DBa and 150ms and 170ms for csl-hdemg. For NinaProDB2 and BioPatRec26MOV, the sliding window steps of test sets are the same as those in existing works [15, 29] which are 100ms and 50ms, respectively.

In previous works on NinaProDB1 and NinaProDB2 [25, 29], the training set consists of approximately 2/3 of the gesture trials of each subject and the remaining trials constitute the test set. For BioPatRec26MOV, we conduct the intra-session cross-validation scheme mentioned in [15]. As each gesture has 3 repetitions in BioPatRec26MOV, the first repetition is applied as the training set and the other two repetitions are applied as the test set [15]. According to previous works on csl-hdemg [26, 27], the intra-session cross-validation scheme was adopted. For each session, a leave-one-out cross-validation is performed, in which each of the 10 trials is used as the test set and the remaining 9 trials are used as training set. For CapgMyo-DBa, the training set consisted of half of the trials, and the other half constitute the test set [26].

Based on the recognition results of test sets, the classification accuracy is calculated for each database as given below:

$$\begin{array}{c} \text{Classification}\;\text{Accuracy}=\frac{\text{Number}\;\text{of}\;\text{correct}\;\text{classifications}}{\text{Total}\;\text{number}\;\text{of}\;\text{test}\;\text{samples}}*100\% \end{array}$$ (11)

### Comparison with existing deep learning approaches

We compare proposed attention-based hybrid CNN-RNN architecture with the state-of-the-art deep learning approaches on five sEMG benchmark databases and the results can be found in Table 4.

**Table 4:** Classification accuracy of the proposed method and previous works.

	NinaProDB1			NinaProDB2		BioPatRec26MOV			CapgMyo-DBa			csl-hdemg		
	150ms	200ms	Trial	200ms	Trial	50ms	150ms	Trial	40ms	150ms	Trial	150ms	170ms	Trial
Feature-LDA [15]	-	-	-	-	-	86.3%	92.9%	-	-	99.0%	-	-	-	-
Traditional-RF [55]	-	75.3%	-	-	-	-	-		-	-	-	-	-	-
AtzoriNet [25]	-	66.6%	-	-	-	-		-	-	-	-	-	-	-
GengNet [26]	-	77.8%	96.7%	-	-	-	-	-	99.0%	99.5%	-	89.3%	90.4%	96.8%
ZhaiNet [29]	-	-	-	78.71%	-	-	-	-	-	-	-	-	-	-
RNN Module with raw-signal	78.1%	79.8%	95.0%	Did not converge		76.4%	82.3%	92.6%	71.8%	80.4%	90.4%	65.3%	71.1%	75.8%
CNN Module with raw-image1	82.6%	83.5%	96.5%	73.4%	97.6%	82.1%	83.9%	92.2%	98.0%	97.7%	98.9%	92.0%	92.1%	95.2%
CNN Module with feature-signal-image1	85.4%	86.3%	97.2%	81.4%	97.5%	85.2%	90.0%	95.8%	-	-	-	-	-	-
Hybrid CNN-RNN with raw-image1	83.5%	84.7%	96.5%	74.6%	97.7%	88.5%	92.2%	96.8%	99.1%	99.6%	99.9%	94.3%	94.8%	96.1%
Hybrid CNN-RNN with feature-signal-image1	86.4%	86.7%	97.1%	82.0%	97.5%	89.9%	93.9%	97.5%	-	-	-	-	-	-
Attention-based hybrid CNN-RNN with raw-image1	83.7%	84.8%	96.5%	74.8%	97.6%	88.7%	92.5%	96.8%	**99.3%**	**99.7%**	**99.9%**	**94.5%**	**94.9%**	**96.1%**
Attention-based hybrid CNN-RNN with feature-signal-image1	**86.8%**	**87.0%**	**97.3%**	**82.2%**	**97.6%**	**90.0%**	**94.1%**	**97.7%**	-	-	-	-	-	-

In this work, the compared approaches are AtzoriNet [25], GengNet [26] and ZhaiNet [29]. We also compare the proposed method with the state-of-the-art traditional machine learning method using a random forest classifier (namely Traditional-RF [55]) and new feature set with LDA classifier (namely Feature-LDA [15]). The proposed architecture on NinaProDB1 using raw-image1 achieves 84.7% classification accuracy of 52 hand gestures which is 6.9% higher than the GengNet. The feature-signal-image1 improves the accuracy from 84.7% to 86.7% which is 8.9% higher than state-of-the-art deep learning approach and 11.4% higher than state-of-the-art traditional machine learning method. The attention mechanism has improved the accuracy from 86.7% to 87.0%. For NinaProDB2, the proposed attention-based hybrid CNN-RNN architecture using feature-signal-image1 achieves 82.2% classification accuracy of 50 hand gestures from 40 subjects which is 78.71% in previous work [29]. The classification accuracy of 26 gestures from BioPatRec26MOV database is 94.1% which is 92.9% in the existing work [15].

The classification accuracy of CapgMyo database is close to saturation, and attention-based hybrid CNN-RNN achieves 99.7% classification accuracy which is 0.2% higher than the GengNet. For csl-hdemg database, the accuracy is improved from 89.3% to 94.5% by the proposed attention-based hybrid CNN-RNN architecture.

After training the attention-based hybrid CNN-RNN model on GPUs, we achieved the trained model which can be applied for sEMG-based gesture recognition on any machine that contains GPU or CPU. In order to discuss the recognition time of each sample for five benchmark databases, we test the trained model on a workstation with one NVIDIA TITAN Xp GPU and one Intel 6850k CPU. The results are shown in Table 5. The recognition time of each sample on GPU is less than 10ms and the model can be applied for prosthetic control and human-computer interaction [61]. The recognition time of each sample on CPU is less than 350ms which satisfies response time constraints for human-computer interaction [61].

**Table 5:** Recognition time of each sample on five benchmark databases with attention-based hybrid CNN-RNN architecture. The recognition window length is 200ms for NinaProDB1 and NinaProDB2, 150ms for BioPatRec26MOV, CapgMyo-DBa and csl-hdemg.

	NinaProDB1	NinaProDB2	BioPatRec26MOV	CapgMyo-DBa	csl-hdemg
GPU	3.0ms	3.6ms	4.1ms	7.8ms	6.0ms
CPU	106ms	140ms	107ms	258ms	327ms

### Ablation studies on the proposed architecture

**Fig 3:**
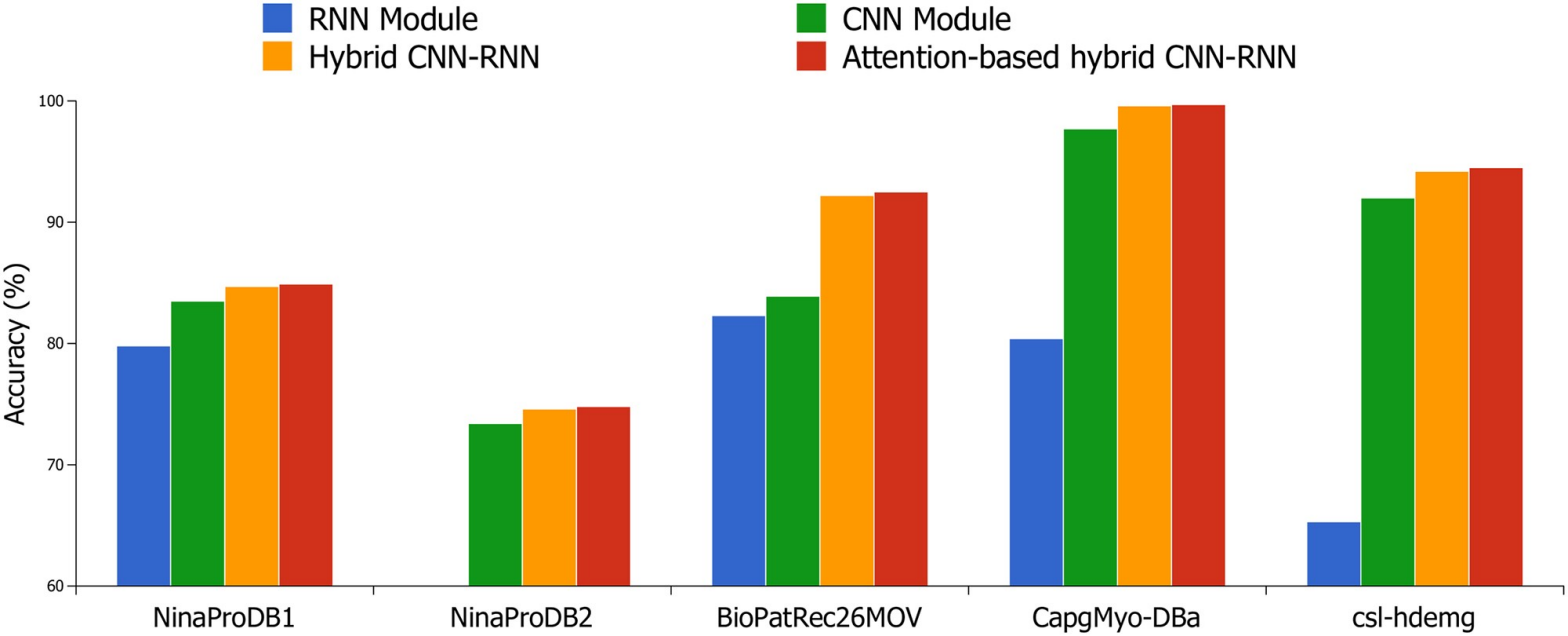
Classification accuracy of RNN module with raw-signal, CNN module hybrid CNN-RNN and attention-based hybrid CNN-RNN architectures with raw-image1 on five benchmark databases.

To prove the advantage of hybrid CNN-RNN, we evaluate the CNN and RNN module which are constructed using model parameters mentioned before. In the CNN module evaluation, the input sEMG data are the same as those in hybrid CNN-RNN, and we employ a softmax layer instead of RNN unit. In the RNN module evaluation, the input is the same as that in hybrid CNN-RNN, but there is no need to convert the electromyogram signal into a sEMG image. Each frame of the input electromyogram signal is directly followed by RNN without extracting high-level features by CNN. Moreover, we inject the attention mechanism into RNN module of proposed hybrid CNN-RNN architecture, and it allows the model to pay attention to the subsegments which contain more discriminative information for gesture recognition. As can be seen in Table 4 and Fig 3, the attention-based hybrid CNN-RNN architecture outperforms the other three frameworks on five sEMG benchmark databases. The improvements of recognition accuracy for attention-based hybrid CNN-RNN architecture are 0.3% on NinaProDB1, 0.2% on NinaProDB2, 0.3% on BioPatRec26MOV, 0.1% on CapgMyo-DBa and 0.2% on csl-hdemg, respectively.

**Fig 4:**
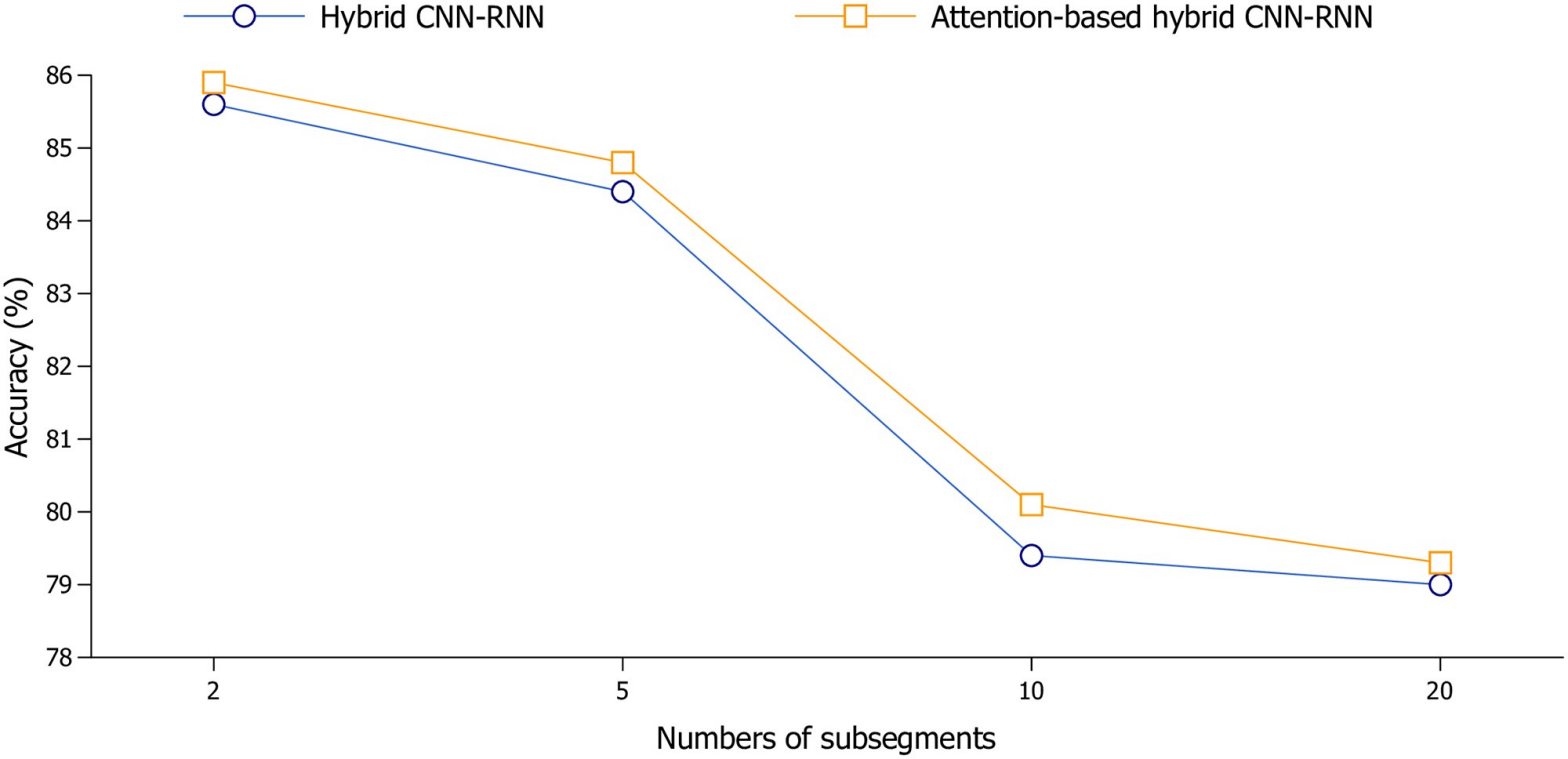
Classification accuracy of attention-based hybrid CNN-RNN architecture with with different numbers of subsegments on NinaProDB1.

Since the accuracy enhancement capability of attention mechanism is influenced by the length of input sequence (i.e., the number of subsegments), we present the results of different numbers of subsegments on NinaProDB1. The number of subsegments is set as {2,5,10,20} and the results are shown in Fig 4. If we set the number of subsegments as 2, the recognition accuracy of the attention-based hybrid CNN-RNN is 0.3% higher than that of the hybrid CNN-RNN. However, if we increase the number of subsegment to 10, accuracy of the attention-based model is 0.7% higher than that of the model without attention.

### Evaluation of different image representation methods

**Fig 5:**
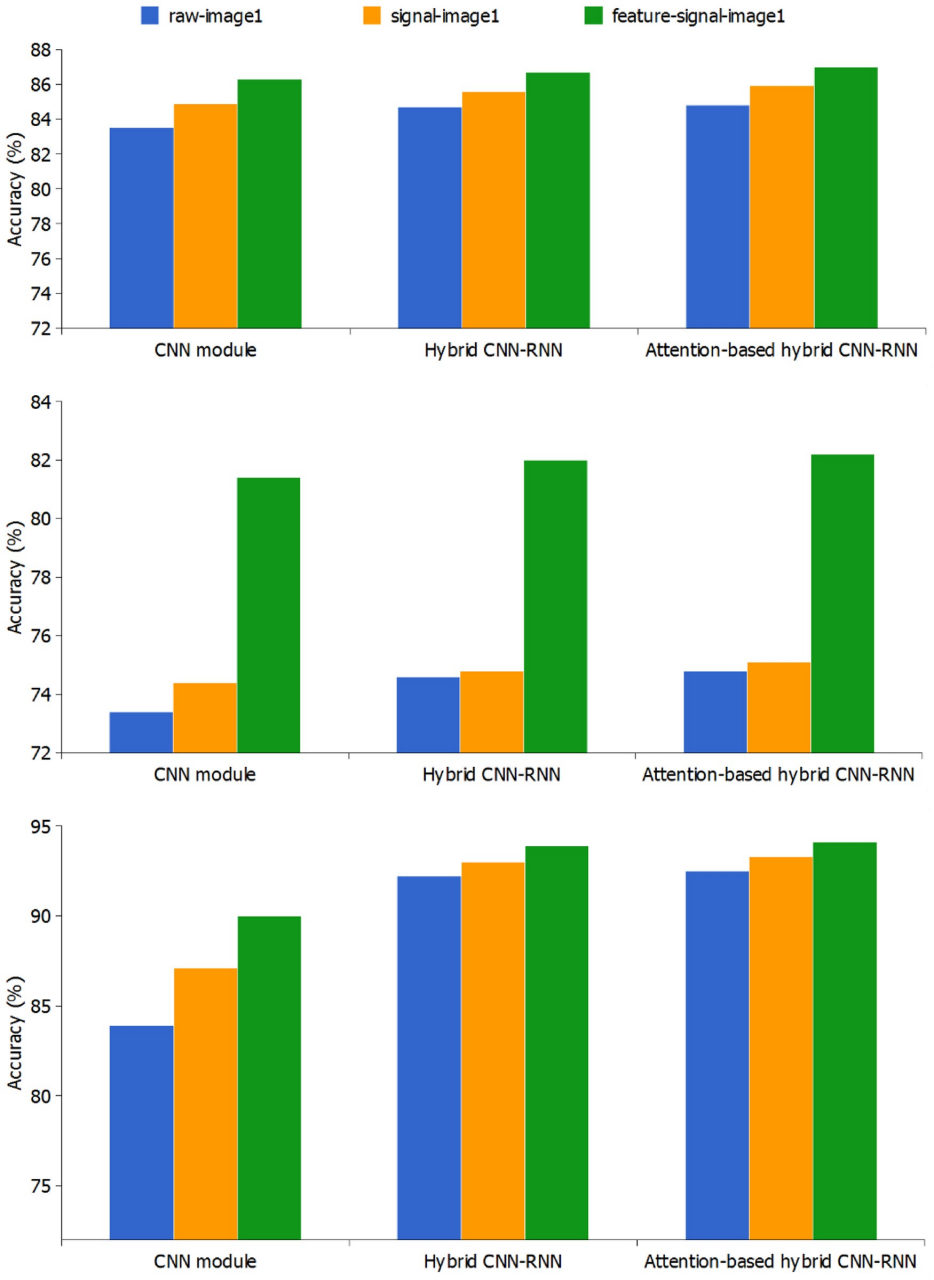
Classification accuracy of CNN module, hybrid CNN-RNN and attention-based hybrid CNN-RNN architectures with three sEMG image representation methods on three sparse multi-channel benchmark databases.

We compare the “feature-signal-image1” with two raw signal based methods “raw-image1” and “signal-image1” for CNN, hybrid CNN-RNN and attention-based hybrid CNN-RNN frameworks. The results in Fig 5 show that the “feature-signal-image1” achieves the best performance for all the three frameworks on three sparse multi-channel databases NinaProDB1, NinaProDB2 and BioPatRec26MOV.

We also evaluate the eight image representation methods on the sparse multi-channel database NinaProDB1 and the results can be seen in Table 6. The raw-image1, raw-image2, signal-image1, signal-image2, activity-image1, activity-image2 and feature-signal-image1 are mentioned in section Methods. The feature-signal-image2 uses the same generation procedure as that of signal-image2 and the raw-image2 has been used in existing work [25]. In Table 6, we find that feature-signal-images formed by feature vectors achieve higher accuracy than sEMG images formed by raw signals. The raw-image1, signal-image1, activity-image1 and feature-signal-image1 obtain higher accuracy than the general image representation methods raw-image2, signal-image2, activity-image2 and feature-signal-image2, respectively. We draw the same conclusion for CNN module, hybrid CNN-RNN and attention-based hybrid CNN-RNN architectures that the **feature-signal-image1** achieves the highest accuracy in the eight evaluated sEMG image representation methods. For the input of RNN module is a vector instead of an image, we also compare the raw signal with feature vector for the RNN module and the accuracies are 79.8% and 74.5%, respectively.

**Table 6:** Classification accuracy of different image representation methods on NinaProDB1. We use the same sliding window length (200ms) for all experiments mentioned bellow.

	CNN Module	hybrid CNN-RNN	Attention-based hybrid CNN-RNN
raw-image1	83.5%	84.7%	84.8%
raw-image2	82.9%	80.8%	80.9%
signal-image1	84.9%	85.6%	85.9%
signal-image2	79.8%	81.6%	82.0%
activity-image1	78.1%	78.8%	79.1%
activity-image2	72.8%	74.1%	74.5%
feature-signal-image1	**86.3%**	**86.7%**	**87.0%**
feature-signal-image2	83.1%	84.5%	84.7%

## Conclusion

In this work, we propose an attention-based hybrid CNN-RNN architecture for sEMG-based gesture recognition, which consists of feature extraction stage and attention-based sequential modeling stage. It makes full use of spatial and temporal information of electromyogram signals and the attention mechanism makes the network more intelligent to pay attention to different parts of the electromyogram signal. The evaluations are performed on five sEMG benchmark databases, namely NinaProDB1, NinaProDB2, BioPatRec26MOV, CapgMyo-DBa and csl-hdemg databases. The results show that 1) the hybrid CNN-RNN architecture outperforms both CNN and RNN modules; 2) the attention mechanism enhances the performance of the hybrid CNN-RNN architecture. Moreover, we present a new feature vector based sEMG image representation method “feature-signal-image1” for sparse multi-channel databases. Compared with the sEMG image representation method “raw-image1”, it improves the recognition accuracy from 84.8% to 87.0% on NinaProDB1, from 74.8% to 82.2% on NinaProDB2, from 92.5% to 94.1% on BioPatRec26MOV. Overall, the recognition accuracies of proposed sEMG-based gesture recognition method are 87.0% for NinaProDB1, 82.2% for NinaProDB2, 94.1% for BioPatRec26MOV, 99.7% for CapgMyo-DBa and 94.5% for csl-hdemg. The improvements are 9.2% (NinaProDB1), 3.5% (NinaProDB2), 1.2% (BioPatRec26MOV), 0.2% (CapgMyo-DBa) and 5.2% (csl-hdemg) higher than the state-of-the-art performances [15, 26, 29, 55].

The electromyogram signal is a kind of biological signal which is severely affected by the difference between subjects. It makes the accuracy of Leave-One-Subject-Out cross-validation (LOSOCV) much lower than that of Within-Subject cross-validation (WSCV) in previous works [14, 27]. Future research will be to improve the accuracy of LOSOCV which is significant for a new user to interact with computers. We will first extend our framework to fuse the sEMG data with IMU data and extract common features of different subjects to improve the LOSOCV accuracy. Then, we will propose a framework to integrate information from various sensors in the HCI system to allow both intact-limbed and amputees to communicate with different kinds of machines efficiently.
